# HOXC6 gene silencing inhibits epithelial-mesenchymal transition and cell viability through the TGF-β/smad signaling pathway in cervical carcinoma cells

**DOI:** 10.1186/s12935-018-0680-2

**Published:** 2018-12-12

**Authors:** Feng Zhang, Chen-Chen Ren, Ling Liu, Yan-Nan Chen, Li Yang, Xiao-An Zhang

**Affiliations:** 1 Department of Obstetrics and Gynecology, The Third Affiliated Hospital of Zhengzhou University, No. 7, Kangfu Front Street, Erqi District, Zhengzhou, 450052 Henan Province People’s Republic of China; 2 Department of Imaging, The Third Affiliated Hospital of Zhengzhou University, No. 7, Kangfu Front Street, Erqi District, Zhengzhou, 450052 Henan Province People’s Republic of China

**Keywords:** HOXC6, TGF-β/smad signaling pathway, Epithelial-mesenchymal transition, Cervical carcinoma, Cell viability

## Abstract

**Background:**

Homeobox C6 (HOXC6) plays a part in malignant progression of some tumors. However, the expression of HOXC6 and its clinical significance remains unclear in cervical carcinoma (CC). The purpose of this study is to verify the effects of HOXC6 gene silencing on CC through the TGF-β/smad signaling pathway.

**Methods:**

CC tissues and corresponding paracancerous tissues were collected from CC patients with involvement of a series of HOXC6-siRNA, HA-HOXC6 and the TGF-β/smad pathway antagonist. HOXC6 expression was analyzed in six CC cell lines (C-33A, HeLa, CaSki, SiHa, ME-180, and HCC-94) by reverse transcription quantitative polymerase chain reaction (RT-qPCR) and western blot analysis. The mRNA and protein expression of HOXC6, TGF-β1, TGF-β RII, smad4, smad7, E-cadherin, N-cadherin, Vimentin, ki-67, proliferating cell nuclear antigen (PCNA), p27, and Cyclin D1 were determined by RT-qPCR and western blot analysis. Cell proliferation, apoptosis and cell cycle were detected by MTT assay and flow cytometry, respectively.

**Results:**

Higher positive expression rate of HOXC6 protein was observed in CC tissues and HOXC6 was related to TNM stage, lymphatic metastasis, cancer types, primary lesion diameter, and histological grade of CC. Silencing HOXC6 inhibited epithelial-mesenchymal transition (EMT) (shown as decreased N-cadherin and Vimentin, and increased E-cadherin) through the inactivation of the TGF-β/smad signaling pathway. HOXC6 gene silencing hindered cell proliferation and accelerated cell apoptosis of CC cells. Furthermore, the effect of HOXC6 silencing was enhanced when the TGF-β/smad signaling pathway was suppressed.

**Conclusion:**

The results reveal that HOXC6 gene silencing may inhibit EMT event and cell viability in CC through the inhibition of the activation of TGF-β/smad signaling pathway.

## Background

Cervical carcinoma (CC) remains the second commonest female cancer across the world and prevalently occurs in many low-income countries, mainly caused by persistent infection with human papilloma virus [1]. Except high incidence, CC is ranked as the 4th most common cancer-related death among women, with 88% of all deaths in developing countries [2]. Additionally, the main symptoms of CC include post-coital vaginal bleeding, post-menopausal vaginal bleeding, intermenstrual vaginal bleeding, lower abdominal pain and offensive vaginal discharge [3]. As a low-cost and simple alternative to traditional Pap smears, visual inspection with acetic acid has been widely used for CC diagnosis, whose advantage is that women can be screened and treated at the same time [4]. Although the diagnostic and therapeutic strategies get improved continuously, the 5-year survival rate for advanced CC patients is still poor [5]. Hence, researches on the potential molecular mechanisms underlying CC progression are urgently needed for effective treatment modalities for CC patients.

Homeobox (HOX) genes, as a family of transcription factors, are responsible for the regulation of gene expression by directly binding to specific DNA sequences of target genes [6]. It has been reported that HOX gene takes part in leukemogenic transformation and is highly expressed in hematologic malignancies [7]. Recent evidence suggests that homeobox C6 (HOXC6) gene, a critical player in milk production and mammary gland development, has been found to be overexpressed in medulloblastomas [8] and osteosarcomas [9], as well as in variety of carcinomas including breast [10], lung [11] and prostate cancer s [12]. A recent study has demonstrated that HOXC6 can exert diagnostic value in early detection and has monitoring function on progression or response to therapy, thus serving as a novel biomarker for prostatic cancer [13]. In addition, HOX proteins are reported to interact with Smads [14]. Smads, as multifunctional proteins, can promote the expression of multiple microRNAs by advancing the cleavage reaction and act as the signal transducers in TGF-β/bone morphogenetic protein pathway [15]. Transforming growth factor-β (TGF-β) signaling pathway plays an essential role in many development processes in human and animals [16], and it has been confirmed as a critical modulator for many cellular processes correlated with carcinogenesis [17]. It has reported that the TGF-β/smad pathway was activated in cervical squamous carcinoma [18]. Based on the above, HOXC6 may play a critical role in CC by binding to the TGF-β/smad signaling pathway. Thus, this study aims to evaluate the mechanism of HOXC6 in relation to the TGF-β/smad signaling pathway in CC. Furthermore, the study defined the hypothesis that HOXC6 gene silencing inhibits epithelial-mesenchymal transition (EMT) through inhibiting the TGF-β/smad signaling pathway in CC.

## Materials and methods

### Ethics statement

This study coincided with the principle of voluntariness with the consent for the volunteers and their family. At the same time, patients had full understanding of the study and the ability to complete all the treatment plans, and signed the relevant informed consent.

### Study subjects and specimens collection

CC tissues and corresponding paracancerous tissues were collected from 72 CC patients without other systemic tumors who were examined pathologically in the Third Affiliated Hospital of Zhengzhou University between January 2014 and January 2017. The age of patients ranged from 29 to 71 years, with an average age of 59.6 ± 6.8 years. All patients were not treated with biotherapy, radiotherapy or chemotherapy before the operation and had complete clinical data. In accordance with the American Joint Committee on Cancer (AJCC) tumor-node-metastasis (TNM) system [19], the CC patients were classified as follows: 56 cases of squamous cell carcinoma, 16 cases of adenocarcinoma; 38 cases in stage I–II, and 34 cases in stage III–IV. Based on the treatment principle of CC, the patient were classified as follows: 44 cases with primary tumor < 4 cm, 28 cases with primary tumor ≥ 4 cm; postoperatively, 43 cases of N0–N1 lymph node metastases, 29 cases of N2–N3 lymph node metastases. All of the specimens were divided into two parts. One was cryopreserved in liquid nitrogen immediately after washing with normal saline and the other was embedded with paraffin and stored after 24 h of fixing with neutral buffered formalin (NBF).

### Immunohistochemistry

The specimens were fixed in formaldehyde, embedded with paraffin, cut into 4 μm serial sections, and baked at 70 °C for 15 min, followed by dehydration with gradient ethanol. After inactivation of endogenous enzyme by H_2_O_2_, the specimens were rinsed with phosphate buffer solution (PBS) 3 times (5 min each time). The sections were incubated with normal goat serum at room temperature for 15 min, and incubated with 20–30 μL HOXC6 primary antibody overnight at 4 °C (diluted 1:50 with PBS; (ab41587; Abcam Inc., Cambridge, MA, USA). PBS was used as a substitute for the primary antibody as a negative control (NC). The sections were rinsed with PBS 3 times, and then incubated with the secondary antibody for 45 min at 37 °C. After being rinsed with PBS, the sections were developed with 3,3-diaminobenzidine (DAB) (Kit-0016; Maixin Biological Technology Development Company, Fuzhou, China) for 5–10 min, and washed with tap water. Next, the sections were counterstained with hematoxylin for 5 min, desalinated by dilute hydrochloric acid for 30 s, and rinsed for 5 min. Subsequently, the sections were dehydrated, cleared, and mounted, followed by examination with a microscopy. The results of immunohistochemistry were examined by 4 senior histopathologists using the double blind method. Cell cytomembrane/cytoplasm stained with light yellow or tan were regarded as positive cells. According to the percentage of positive cells, positive cells/total cells ≤ 5% was scored as 0 point, positive cells/total cells 6–25% as 1 point, positive cells/total cells 26–50% as 2 points, and positive cells/total cells > 50% as 3 points. According to the staining intensity of immunohistochemistry, negative was scored as 0 point, light yellow as 1 point, moderate yellow as 2 points, and tan as 3 points. The judgment of protein expression is based on both the staining intensity and positive cell rate, and the product of these two values was calculated. Positive group was defined as the product was ≥ 3 points, while negative group was defined as the product < 3 points.

### Cell culture and selection

The human CC cell lines C-33A, HeLa, CaSki, SiHa, ME-180, and HCC-94 were purchased from Shanghai Institutes for Biological Sciences, Chinese Academy of Sciences (Shanghai, China). C-33A, CaSki and HCC-94 cell lines were cultured in a RPMI 1640 medium (Gibco™; Cat.No.71260, Invitrogen, Carlsbad, CA, USA), ME-180 cells in McCoy’S 5A medium (Gibco™; Cat.No.16600, Invitrogen, Carlsbad, CA, USA), and HeLa and SiHa cells in McCoy’s 5A medium (Gibco™; Cat.No.727635, Invitrogen, Carlsbad, CA, USA). All mediums were added with 10% fetal bovine serum (FBS) and 10^4^ μ/L penicillin/streptomycin for cell subculture. The cells were passaged every 2–3 days in 5% CO_2_ incubators at 37 °C. The cells were passaged to the third to fifth generation, and cells at the logarithmic growth phase were selected for subsequent experiments. Reverse transcription quantitative polymerase chain reaction (RT-qPCR) was used to determine the expression of HOXC6 in different CC cell lines, and 2 cell lines with the highest expression were selected for the follow-up experiment. The experiment was repeated three times.

### Plasmid construction

The effectiveness of siRNA targeting different loci of different genes was distinct. In accordance with the principle of complementarity proposed by Reynolds and Elbashir et al., siRNA sequences were designed and synthesized by Shanghai GenePharma Co., Ltd. (Shanghai, China) and listed as follows: control siRNA: UUCUCCGAACGUGUCACGUDTDT (forward) and ACGUGACACGUUCGGAGAADTDT (reverse) and three HOXC6 siRNA sequences: HOXC6-siRNA-1: GUCCCUAUAACCAUCUAGUDTDT (forward) and ACUAGAUGGUUAUAGGGACDTDT (reverse); HOXC6-siRNA-2: CUUCUUGCAACUCUCCUUADTDT (forward) and UAAGGAGAGUUGCAAGAAGDTDT (Reverse); HOXC6-siRNA-3: CCGUAUGACUAUGGAUCUADTDT (forward) and UAGAUCCAUAGUCAUACGGDTDT (reverse). Conventionally, the cells were transduced with pQCXIH-HA empty plasmid, pQCXIH-HA-HOXC6 retrovirus plasmid, and corresponding package plasmids (VSVG and GPZ). After 72 h, the supernatant was collected, processed by viral titer identification, sub-packed and preserved at − 80 °C. The segment of short hairpin RNA (shRNA) with complementary sequence of HOXC6 was cloned into pLKO.1 lentiviral vector (Addgene Company, Cambridge, MA, USA), and confirmed by restriction enzyme XbaI and DNA sequencing analysis. The CC cells were transduced with lentiviral vector scramble shRNA and corresponding package plasmids (VSVG and dv8.2) using FuGENE6 transfection reagents (plasmid:FuGENE6 = 1 μg:3 μL; Promega Corp., Madison, Wisconsin, USA). After 48 h, the supernatant was collected, processed by viral titer identification, sub-packed and preserved at − 80 °C. Subsequently, C3H10T1/2 cells were transduced for 12 h with pQCXIH-HA and pQCXIH-HA-HOXC6 retrovirus using 6 μg/mL polybrene (Sigma-Aldrich Chemical Company, St Louis, MO, USA). After 48 h, stable-transduced cells with pQCXIH-HA and pQCXIH-HA-HOXC6 were obtained after 200 μg/mL hygromycin screening for 7 days. Western blot analysis was used to detect the expression of HA-HOXC6 at the protein level.

### Cell transduction and grouping

Cells at the logarithmic phase were seeded onto a 6-well plate and incubated in a fresh complete medium until cell confluence reached to 50–80%. Subsequently, lipofection was performed in line with the instructions of Lipofectamine 2000 (11668-027; Invitrogen Inc., Carlsbad, CA, USA). Briefly, 200 μL complex of transfection reagents and plasmids/vectors was prepared and cell transduction was carried out under serum-cultured conditions in 5% CO_2_ at 37 °C for 6 h. The cells were assigned into: blank (human CC cells), NC (human CC cells + empty vector), HA-HOXC6 (human CC cells + HOXC6 overexpression vector), HOXC6-siRNA (human CC cells + HOXC6-siRNA vector), SB431542 (human CC cells + TGF-β/smad pathway antagonist), SB431542 + HOXC6-siRNA (human CC cells + TGF-β/smad pathway antagonist + HOXC6-siRNA vector), and HA-TGF-β1 groups (the overexpressed plasmid of TGF-β1). After transduction, the cells were starved for 24 h. The experiment was repeated three times.

### Reverse transcription quantitative polymerase chain reaction (RT-qPCR)

Total RNA was extracted from CC tissues and paracancerous tissues or cells after treatment using Trizol agent (TaKaRa Biotechnology Co., Ltd., Dalian, Liaoning China) and its purity and concentration were determined. Next, reverse transcription of RNA samples (10 μL) was conducted in order to prepare complementary DNA (cDNA) according to the instructions of reverse transcription kit (DRR047S; TaKaRa Biotechnology Co., Ltd., Dalian, Liaoning China). The obtained cDNA was diluted in 65 μL diethyl phosphorocyanidate (DEPC) and fully mixed. RT-qPCR was conducted with following reaction system: 5 μL SsoFast EvaGreen Supermix (170882; Bio-Rad, Inc., Hercules, CA, USA), 0.5 μL forward primer (10 μM), 0.5 μL reverse primer (10 μM), and 4 μL cDNA. The reaction conditions were pre-denaturation at 95 °C for 1 min, 30 cycles of denaturation at 95 °C for 30 s, annealing at 58 °C for 5 s, and extension at 72 °C for 5 s. The primers were synthesized by Shenzhen BGI Health Technology Co., Ltd. (Shenzhen, China) (Table 1). Glyceraldehyde-3-phosphate dehydrogenase (GAPDH) was taken as the internal reference and genes in each sample were set for three repetitions. The reliability of PCR results was evaluated by solubility curve: ∆Ct = CT _(target gene) _− CT _(internal reference)_, ∆∆Ct = ∆Ct _(experimental group) _− ∆Ct _(control group)_. The relative expression of related genes were obtained by 2^−ΔΔCT^ [20], and the experiment was repeated three times.

**Table 1:** The primer sequences for RT-qPCR

Name	Sequence (5′–3′)
HOXC6	Forward: CTCCACGGTGACTGGAAGTG
	Reverse: AGCTGCGGTAGTCATCATGC
TGF-β1	Forward: GATACGGAGTAACGTCGGGG
	Reverse: CATTCCCTTGGAGCCAGGTC
TGF-βR II	Forward: AGCAACTGCAGCATCACCTC
	Reverse: TGATGTCTGAGAAGATGTCC
smad4	Forward: TGCTGGCATCGGTGTAGATGA
	Reverse: TAGGGCATCCATGGTGAGAGA
smad7	Forward: TTCCTCCGCTGAAACAGGG
	Reverse: CCTCCCAGTATGCCACCAC
E-cadherin	Forward: GTGTTCGCTATTGGACGGGA
	Reverse: TCATAACAGCCGTACCTGGC
N-cadherin	Forward: CTGTCTGGAAAACACCGAGC
	Reverse: TTTCTGCTCCCGCCACAAA
Vimentin	Forward: GCGCGATGTTCGTCAACAAT
	Reverse: TACTCACGAAGATGGCGAGC
GAPDH	Forward: CACCAGGTTGTGTCCACTGA
	Reverse: CCAGATGGGAGAATGGTCGC

*RT-qPCR* reverse transcription quantitative polymerase chain reaction, *GAPDH* glyceraldehyde-3-phosphate dehydrogenase, *HOXC6* homeobox C6, *TGF-β*, transforming growth factor-β/smad signaling pathway

### Western blot analysis

Human CC tissues and paracancerous tissues were placed in centrifuge tubes and added with lysate in 1:8 (m/V). The tissues were intermittently homogenized by ice bath 6 times (6 s each time) and centrifuged at 27,000×*g* for 5 min, and the supernatant was extracted as the total protein. Total protein was divided into two parts: one part was used for determination of protein concentration; the other was added with appropriate 5× loading buffer, mixed, bathed in boiling water for 5 min, and preserved at − 80 °C. Equal amounts of total protein were transferred onto polyvinylidene fluoride (PVDF) membrane after separation by sodium dodecyl sulfate polyacrylamide gel electrophoresis (SDS-PAGE). The membrane was washed once with tris buffered saline with Tween 20 (TBST), blocked with 5% non-fat milk powder, and shaken for 2 h. The membrane was washed three times with TBST and then incubated with primary antibodies diluted by sealing fluid, including HOXC6 (1:2000, AB151575), TGF-β1 (1:2000, ab27969), TGF-β RII (1:1000, ab61213), smad4 (1:2000, ab40759), smad7 (1:1000, ab90086), E-cadherin (1:10000, ab40772), N-cadherin (1:1000, ab76057), Vimentin (1:2000, ab92547), ki-67 (1:1000, ab16667), proliferating cell nuclear antigen (PCNA) (1:1000, ab18197), p27 (1:5000, ab32034), Cyclin D1 (1:10000, ab134175), and GAPDH (1:2500, ab9458) overnight at 4 °C. All above antibodies were purchased from Abcam Inc. (Cambridge, MA, USA). The membrane was returned to room temperature by a shaker, washed with TBST 3 times, and then incubated with horse radish peroxidase (HRP)-labeled secondary antibody (1:5000) at room temperature for 2 h. Next, the membrane was washed with TBST three times, and imaged using the enhanced chemiluminescence (ECL) imaging system (WD-9413A, Beijing Liuyi Instrument Factory, Beijing, China). Gray value was determined by Quantity One software (Bio-Rad Inc., Hercules, CA, USA). The ratio of the gray values of the target genes to the internal reference was considered to be the expression of target proteins. The experiment was repeated three times.

### 3-(4, 5-dimethylthiazol-2-yl)-2, 5-diphenyltetrazolium bromide (MTT) assay

After transduction, the cells at logarithmic growth phase were diluted into single cell suspension and cell concentration was adjusted to 2.0 × 10^7^ cells/L. Then, the cell suspension was transferred into 96-well plates with 100 μL each well. Three groups were set for each cell line and three repetitions were set in each group with 5 × 10^3^ cells in each well. The cells was incubated in a CO_2_ incubator at 37 °C for 24 h, 48 h, 72 h and 96 h, and each well was added with 10 μL MTT solution (Sigma-Aldrich Chemical Company, St Louis, MO, USA) in the dark for incubation for 4 h. After extraction of culture supernatant, each well was added with 100 µL Dimethyl Sulphoxide (DMSO) in the dark. The plate was oscillated on a flat plate oscillator for 15 min to fully dissolve the DMSO, and the optical density (OD) of each well was read at wavelength of 570 nm in a Microplate Reader (BioTek, Winooski, VT, USA). Cell viability curve was drawn with time as X-axis and OD value as Y-axis. The experiment was repeated three times.

### Propidium iodide (PI) staining

After 48-h transduction, cells at logarithmic growth phase were collected to prepare 5 × 10^6^ cells/L of single cell suspension. The cells were rinsed with PBS, resuspended in 70% ethanol and PBS containing 0.5 mmoL LEDTANa_2_, and fixed at 4 °C for 1 h. Subsequently, the cells were centrifuged at 300×*g*, suspended with mixture of 0.01% Trition X-100 and 50 g/L PBS, transferred into a testing tube, and then stained with 0.5 g/L PI dye liquor for 30 min in the dark. Flow cytometer (Coulter Corporation, San Diego, California, USA) was used for cell counting, and the percentage of cells in each phase was observed. The experiment was repeated three times.

### Annexin V-FITC/PI double staining

At 48 h after transduction, the cells in each group were treated with ethylene diamine tetraacetic acid (EDTA)-free trypsin and placed in flow tubes (a few dead cells and apoptotic cells were included in the supernatant and all of them were involved in the statistics), followed by centrifugation at 100×*g* for 5 min. After discarding the supernatant, the cells were washed with cold PBS 3 times, with the supernatant removed. According to the protocol of Annexin-V-FITC kit (Sigma-Aldrich Chemical Company, St Louis, MO, USA), each tube was added 150 μL binding buffer and 5 μL Annexin V-FITC, mixed gently, and incubated at room temperature for 15 min in the dark. Each tube was added with 100 μL binding buffer and 5 μL PI (Sigma, Saint Louis, Missouri, USA), and mixed. The control group was not added with Annexin V as a correction factor. Cell apoptosis was detected within 1 h by flow cytometry (Coulter Corporation, San Diego, California, USA). The results were shown by a four lattice graph with right upper limit as the proportion of advanced apoptotic cells, right lower limit as the proportion of early apoptotic cells, left upper limit as the proportion of dead cells, left lower limit as the proportion of living cells. The experiment was repeated three times.

### Statistical analysis

All data was analyzed using SPSS Version 22.0 software (IBM Corporation, San Diego, CA, USA). The measurement data with normal distribution was exhibited as the mean ± standard deviation (SD). Comparison between CC tissues and paracancerous tissues was conducted using paired *t* test and pairwise comparison between the other groups using student’s *t*-test. The comparisons among multiple groups were conducted by the one-way analysis of variance (ANOVA) for variance analysis and significance test. The data at different time points were compared using repeated-measurement ANOVA. Pearson correlation analysis was used to analyze the linear interaction of HOXC6 mRNA to TGF-β1, TGF-β RII, smad4, and smad7 mRNA. The enumeration data were represented as percentage and analyzed by chi-square test. *p* < 0.05 was considered to be of statistically significance.

## Results

### HOXC6 is upregulated and the TGF-β/smad4 signaling pathway is activated in CC

**Fig. 1:**
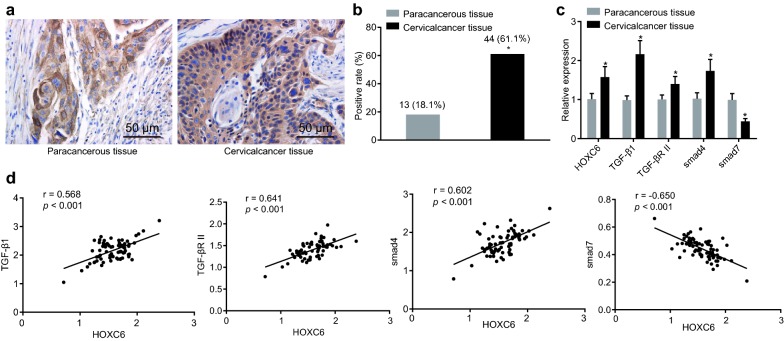
Overexpressed HOXC6 contributes to the occurrence of CC. **a** Positive expression of HOXC6 protein in CC tissues and paracancerous tissues (× 200); scale bar = 50 μm; **b** positive rate of HOXC6 protein between in CC and paracancerous tissues; **c** relative expression of HOXC6, TGF-β1, TGF-βRII, smad4, and smad7 in CC tissues (n = 44) and paracancerous tissues (n = 13) detected by RT-qPCR; **d** linear interaction of HOXC6 to TGF-β1, TGF-β R II, smad4, and smad7 analyzed using Pearson correlation analysis; n = 72; *p < 0.05 compared with paracancerou tissues. *CC* cervical carcinoma, *HOXC6* homeobox C6, *RT-qPCR* reverse transcription quantitative polymerase chain reaction, *TGF* transforming growth factor, *smad* solvated metal atom dispersion

Immunohistochemistry was used to determine positive expression rate of HOXC6 to analyze the function of HOXC6 in CC. As shown in Fig. 1a, b, HOXC6 protein was expressed in the cytoplasm, and positive cells displayed granules in light yellow to tan, which scattered or clustered together in cytoplasm. The results of immunohistochemistry showed that: the HOXC6 protein was found in 44 CC tissue samples, with the positive rate of 61.1% and the staining intensity of 3 points; the HOXC6 protein was found in 13 paracancerous tissue samples, with the positive rate of 18.1% and the staining intensity of 1 point. The positive expression rate of HOXC6 protein in CC tissues was significantly higher than that in paracancerous tissues (*p* < 0.05). Results of RT-qPCR, which was performed to detect the mRNA expression of HOXC6, TGF-β1, TGF-β RII, smad4, and smad7, suggested that when compared to the paracancerous tissues, the CC tissues presented upregulated mRNA expression of HOXC6, TGF-β1, TGF-βRII, smad4, but downregulated that of smad7 (all *p* < 0.05; Fig. 1c). The linear interaction of HOXC6 to TGF-β1, TGF-β RII, and smad4 was analyzed using Pearson correlation analysis, finding that HOXC6 was positively correlated to TGF-β1, TGF-β RII, and smad4 but negatively correlated to smad7 (all *p* < 0.05; Fig. 1d). Thus, CC tissues exhibit elevated HOXC6 expression and the activated TGF-β/smad4 signaling pathway and HOXC6 expression is positively correlated to the TGF-β/smad4 signaling pathway.

### Expression of HOXC6 is associated with the severity of the development of CC

Subsequently, the association between HOXC6 protein and clinicopathological characteristics in CC patients was analyzed in this study. The results revealed that HOXC6 protein expression was related to TNM stage, lymphatic metastasis, cancer types, primary lesion diameter and histological grade (all *p* < 0.05), but had no correlation with age (*p* > 0.05) (Table 2). Therefore, HOXC6 expression is associated with severity of the development of CC.

**Table 2:** Relationship between HOXC6 protein and clinicopathological characteristics in CC patients

Influence factors	Cases	HOXC6		*p* value
		Positive	Negative	
Age (years)				0.427
< 55	51	33	18	
≥ 55	21	11	10	
TNM stage				0.016
I–II	38	18	20	
III–IV	34	26	8	
Lymphatic metastasis				0.001
N0–N1	43	19	24	
N2–N3	29	25	4	
Cancer type				0.019
Squamous cell carcinoma	56	30	26	
Sdenocarcinoma	16	14	2	
Primary lesion diameter (cm)				0.006
< 4	44	21	23	
≥ 4	28	23	5	
Histological grade				0.016
G1–G2	41	20	21	
G3	31	24	7	

*CC* cervical carcinoma, *HOXC6* homeobox C6. Chi-square test was used for statistical analysis

### Higher HOXC6 expression is found in HeLa and SiHa cell lines

**Fig. 2:**
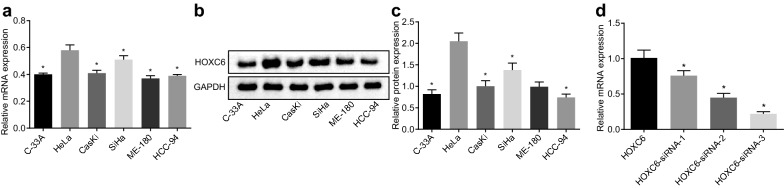
Higher HOXC6 expression is found in HeLa and SiHa cell lines. **a** Relative mRNA expression of HOXC6 in six human CC cell lines; **b** gray value of HOXC6 protein band in six human CC cell lines; **c** HOXC6 protein expression in six human CC cell lines; *p < 0.05 compared with HeLa cells; **d** relative expression of HOXC6, HOXC6-siRNA-1, HOXC6-siRNA-2, and HOXC6-siRNA-3; *p < 0.05, compared with HOXC6; *GAPDH* glyceraldehyde-3-phosphate dehydrogenase, *HOXC6* homeobox C6, *RT-qPCR* reverse transcription quantitative polymerase chain reaction

To explore which cell line is more suitable for our experiment, RT-qPCR and western blot analysis were performed to measure HOXC6 expression in C-33A, HeLa, CaSki, SiHa, ME-180 and HCC-94 cell lines. The results (Fig. 2a–c) showed that: Hela cells showed the highest HOXC6 mRNA expression followed by SiHa cells, and the rest cell lines had no significant difference in HOXC6 mRNA expression. Furthermore, the results of western blot analysis were consistent with HOXC6 mRNA expression in six kinds of human CC cells. Due to higher expression of HOXC6 in HeLa and SiHa cell lines, these two cell lines were chose for the subsequent experiments. Since the effectiveness of siRNA targeting different loci of different genes was distinct, this study designed three different siRNA sequences to analyze their interference efficiency, finding that HOXC6-siRNA-3 had the best interference efficiency and was selected for subsequent experiments (Fig. 2d).

### HOXC6 gene silencing inhibits the activation of TGF-β1/smad signaling pathway in CC cells

**Fig. 3:**
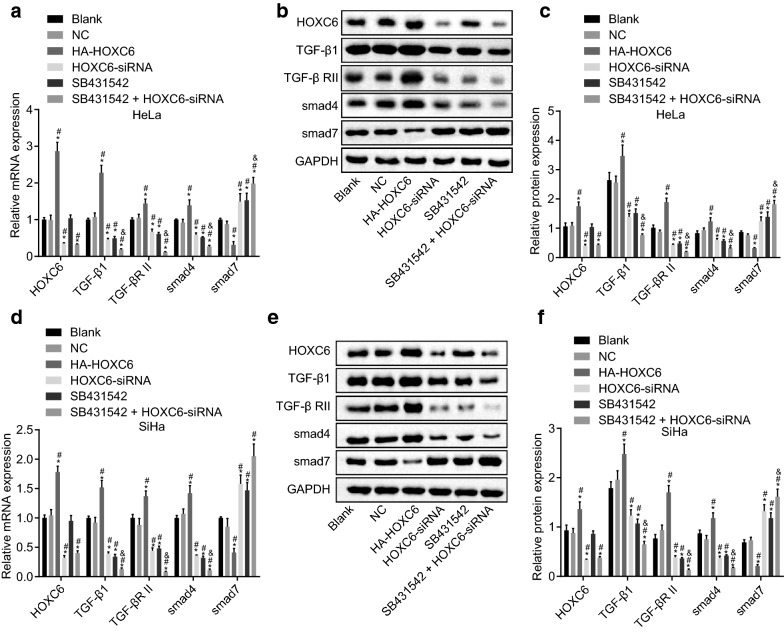
HOXC6 gene silencing inhibits the activation of the TGF-β1/smad signaling pathway in CC cells. **a** mRNA expression of HOXC6, TGF-β1, TGF-β RII, smad4 and smad7 in HeLa cells; **b** gray values of HOXC6, TGF-β1, TGF-β RII, smad4, smad7, and GAPDH in HeLa cells; **c** protein expression of HOXC6, TGF-β1, TGF-β RII, smad4 and smad7 in HeLa cells; **d** mRNA expression of HOXC6, TGF-β1, TGF-β RII, smad4 and smad7 in SiHa cells; **e** gray values of HOXC6, TGF-β1, TGF-β RII, smad4, smad7, and GAPDH in SiHa cells; **f** protein expression of HOXC6, TGF-β1, TGF-β RII, smad4 and smad7 in SiHa cells; *p < 0.05 compared with the blank group; ^#^*p* < 0.05, compared with the NC group; ^&^*p* < 0.05, compared with the SB431542 group. *NC* negative control, *GAPDH* glyceraldehyde-3-phosphate dehydrogenase, *HOXC6* homeobox C6, *TGF-β* transforming growth factor-β, *smad* solvated metal atom dispersion, *RT-qPCR* reverse transcription quantitative polymerase chain reaction

To explore the effect of HOXC6 on the TGF-β1/smad signaling pathway, the mRNA and protein expression of HOXC6, TGF-β1, TGF-β RII, smad4 and smad7 were determined by RT-qPCR and western blot analysis. As shown in Fig. 3a–f, compared with the blank group, expression of HOXC6, TGF-β1, TGF-β RII, and smad4 were significantly decreased but smad7 was markedly increased in the HOXC6-siRNA, SB431542 and SB431542 + HOXC6-siRNA groups, which was opposite with the tendency in the HA-HOXC6 group (all *p* < 0.05). Compared with the HOXC6-siRNA and SB431542 groups, the SB431542 + HOXC6-siRNA group showed significantly decreased mRNA and protein expression of HOXC6, TGF-β1, TGF-β RII, and smad4 (all *p* > 0.05). There was no significant difference in expression of HOXC6, TGF-β1, TGF-β RII, smad4 and smad7 between the NC and blank groups, or between the HOXC6-siRNA and SB431542 groups (all *p* > 0.05). In SiHa cells, expression of HOXC6, TGF-β1, TGF-β RII, smad4 and smad7 were identical to that in HeLa cells, but the expression of those genes were weaker than that in HeLa cells. It indicated that HOXC6 siRNA could suppress the activation of TGF-β1/smad signaling pathway in CC cells.

### HOXC6 gene silencing inhibits EMT in CC cells

**Fig. 4:**
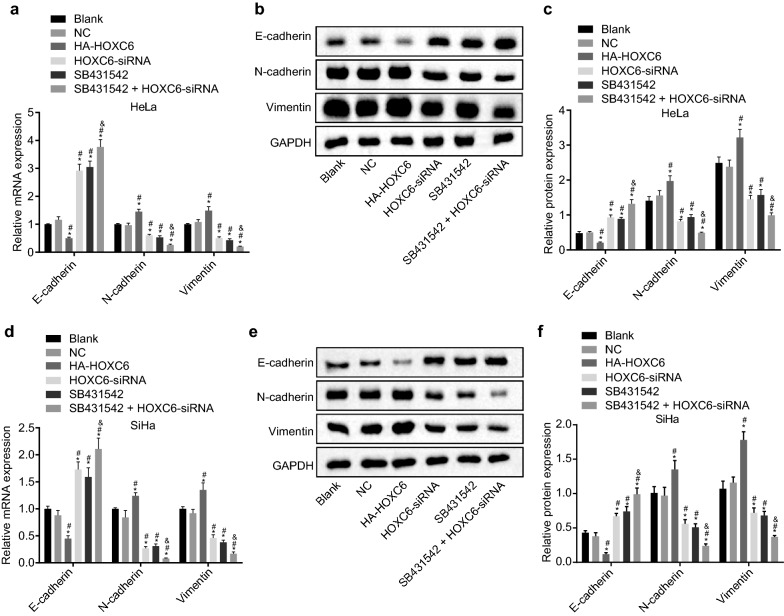
HOXC6 gene silencing suppresses EMT in CC cells. **a** mRNA expression of E-cadherin, N-cadherin and Vimentin in HeLa cells; **b** gray values of E-cadherin, N-cadherin, Vimentin, and GAPDH in HeLa cells; **c** protein expression of E-cadherin, N-cadherin and Vimentin in HeLa cells; **d** mRNA expression of E-cadherin, N-cadherin and Vimentin in SiHa cells; **e** gray values of E-cadherin, N-cadherin, Vimentin, and GAPDH in SiHa cells; **f** protein expression of E-cadherin, N-cadherin and Vimentin in SiHa cells; *p < 0.05 compared with the blank group; ^#^*p* < 0.05, compared with the NC group; ^&^*p* < 0.05, compared with the SB431542 group. *NC* negative control, *GAPDH* glyceraldehyde-3-phosphate dehydrogenase, *HOXC6* homeobox C6, *TGF-β* transforming growth factor-β, *smad* solvated metal atom dispersion, *RT-qPCR* reverse transcription quantitative polymerase chain reaction

Twenty-four hours after transduction with HOXC6-siRNA in HeLa and SiHa cell lines, RT-qPCR and western blot analysis were used to determine the mRNA and protein expression of E-cadherin, N-cadherin and Vimentin to investigate how HOXC6 affected EMT in CC cells. Compared with the blank group, the expression of N-cadherin and Vimentin were remarkably increased in the HA-HOXC6 group, while decreased in the HOXC6-siRNA, SB431542, and SB431542 + HOXC6-siRNA groups; E-cadherin expression was notably decreased in the HA-HOXC6 group, while increased in the HOXC6-siRNA, SB431542, and SB431542 + HOXC6-siRNA groups (all *p* < 0.05). The change of each index in the HOXC6-siRNA group was contrary to that in the HA-HOXC6 group (*p* < 0.05). In comparison to the HOXC6-siRNA and SB431542 groups, the SB431542 + HOXC6-siRNA group showed a significant decrease in the expression of N-cadherin and Vimentin and a significant increase in E-cadherin expression (all *p* < 0.05) (Fig. 4a–f). These findings demonstrated that HOXC6 siRNA could upregulate E-cadherin expression and downregulate N-cadherin and Vimentin expression in HeLa and SiHa cells.

### HOXC6 gene silencing inhibits cell proliferation in CC cells

**Fig. 5:**
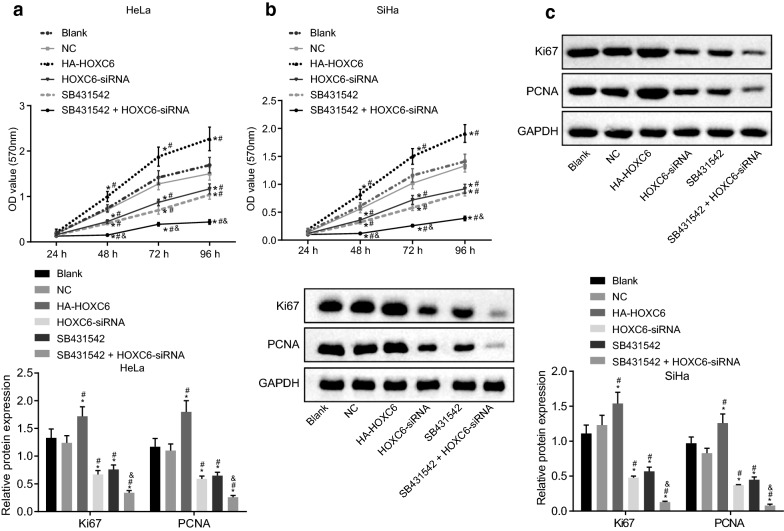
HOXC6 gene silencing inhibits cell proliferation in CC cells. **a** HeLa cell proliferation after transduction; **b** SiHa cell proliferation after transduction; **c** relative protein expression of Ki67 and PCNA in each cell line determined by western blot analysis; *p < 0.05 compared with the blank group; ^#^*p* < 0.05, compared with the NC group; ^&^*p* < 0.05, compared with the SB431542 group; *NC* negative control, *OD* optical density, *MTT*, 3-(4, 5-dimethylthiazol-2-yl)-2, 5-diphenyltetrazolium bromide, *HOXC6* homeobox C6, *PCNA* proliferating cell nuclear antigen

To explore the effect of HOXC6 on cell proliferation, MTT assay was performed to detect cell viability in HeLa and SiHa cells. The cell viability in each group showed an upward trend at every time point, and kept consistent in HeLa and SiHa cell lines. Compared with the blank group, cell proliferation was significantly promoted in the HA-HOXC6 group, while suppressed in the HOXC6-siRNA, SB431542, and SB431542 + HOXC6-siRNA groups at each time point after 48 h of transduction (all *p* < 0.05). In comparison to the HOXC6-siRNA and SB431542 groups, the SB431542 + HOXC6-siRNA group showed an inhibition of cell proliferation (*p* < 0.05). There was no significant difference in cell viability in HeLa and SiHa cells between the NC and blank groups or between the HOXC6-siRNA and SB431542 groups (all *p* > 0.05). The relative protein expression of Ki67 and PCNA in each cell line was determined using western blot analysis, showing that in contrast to the NC and blank groups, the relative protein expression of Ki67 and PCNA was overexpressed in the HA-HOXC6 group but decreased in the HOXC6-siRNA, SB431542, and SB431542 + HOXC6-siRNA group (Fig. 5a–c). These results suggested that HOXC6 siRNA had inhibitory effect on the proliferation in HeLa and SiHa cells.

### HOXC6 gene silencing inhibits cell cycle progression of CC cells

**Fig. 6:**
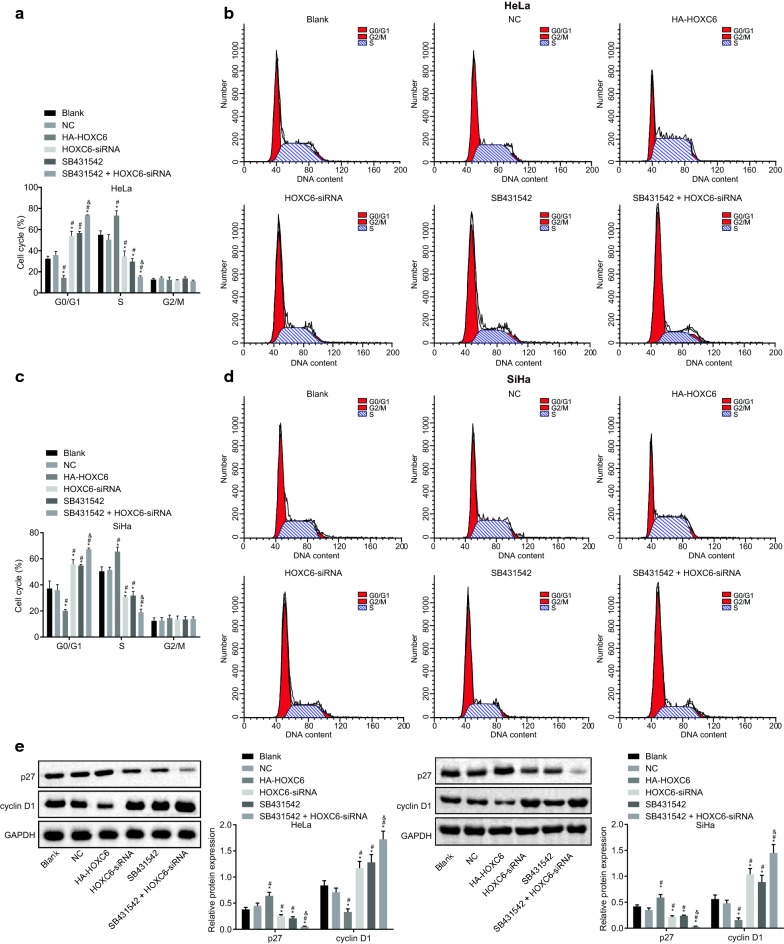
HOXC6 gene silencing arrests cells in G0/G1 phase in CC cells. **a** Cell cycle of each group in HeLa cells; **b** cell cycle of each group in SiHa cells; **c** cell cycle distribution in HeLa cells; **d** cell cycle distribution in SiHa cells; **e** relative protein expression of p27 and cyclin D1 in each cell line determined by western blot analysis; *p < 0.05 compared with the blank group; ^#^*p* < 0.05, compared with the NC group; ^&^*p* < 0.05, compared with the SB431542 group; *NC* negative control, *PI* propidium iodide, *HOXC6*, homeobox C6

To explore how HOXC6 affect cell proliferation, PI staining was carried out to detect the cell cycle distribution in HeLa and SiHa cells after transduction. The cell cycle in each group showed consistent trend in HeLa and SiHa cell lines (Fig. 6a–d). Compared with the blank group, the HOXC6-siRNA and SB431542 groups showed higher percentage of cells at G0/G1 phase and lower percentage of cells at S phase (all *p* < 0.05). In comparison to the blank and NC groups, the HA-HOXC6 group showed lower percentage of cells at G0/G1 phase and higher percentage of cells at S phase, while the SB431542 + HOXC6-siRNA group showed higher percentage of cells at G0/G1 phase and lower percentage of cells at S phase (*p* < 0.05). There were no significant differences of cell cycle distribution between the NC and blank groups, as well as the HOXC6-siRNA and SB431542 groups (*p* > 0.05). Western blot analysis was carried out to detect the relative protein expression of p27 and cyclin D1 in each cell line. The results (Fig. 6e) revealed that in comparison with the blank and NC groups, the protein expression of p27 was elevated in the HA-HOXC6 group, but reduced in the HOXC6-siRNA, SB431542, and SB431542 + HOXC6-siRNA groups. The protein expression of cyclin D1 was opposite to that of p27. These results suggested that downregulation of HOXC6 could significantly inhibit the proliferation in HeLa and SiHa cells.

### HOXC6 gene silencing promotes cell apoptosis in CC cells

**Fig. 7:**
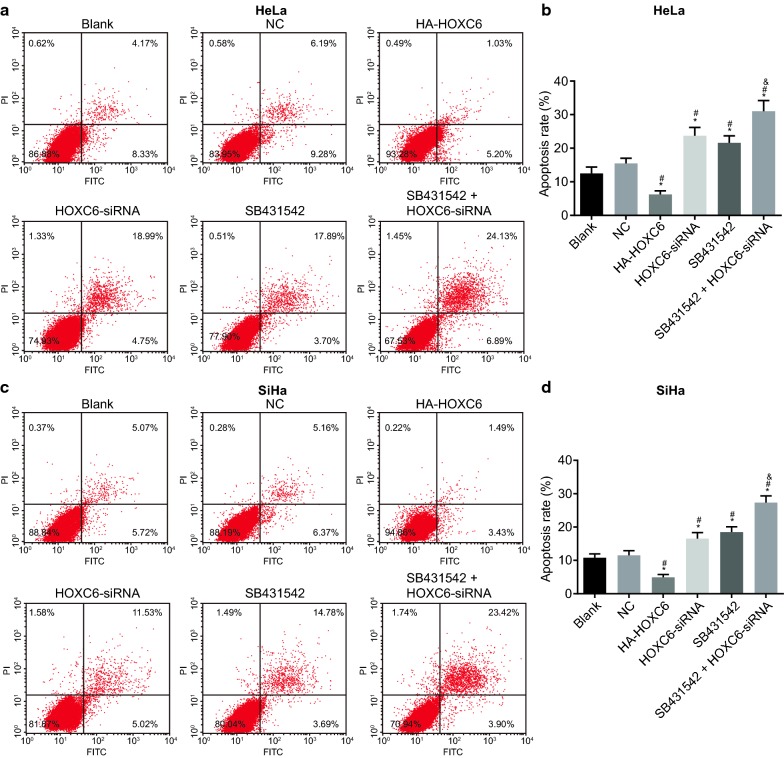
HOXC6 gene silencing promotes cell apoptosis in CC cells. **a** HeLa cell apoptosis after transduction; **b** apoptosis rate of HeLa cells after transduction; **c** SiHa cell apoptosis after transduction; **d** apoptosis rate of SiHa cells after transduction; *p < 0.05 compared with the blank group; ^#^*p* < 0.05, compared with the NC group; ^&^*p* < 0.05, compared with the SB431542 group; *NC* negative control, *HOXC6* homeobox C6

Cell apoptosis was tested by flow cytometry. In comparison to the blank group, cell apoptosis rate was significantly decreased in the HA-HOXC6 group and increased in the HOXC6-siRNA, SB431542, and SB431542 + HOXC6-siRNA groups (all *p* < 0.05). There was no statistical significance between the NC and blank groups, as well as the HOXC6-siRNA and SB431542 groups (*p* > 0.05) (Fig. 7). The above findings indicated that HOXC6 gene silencing could induce cell apoptosis of CC cells.

### HOXC6 silencing represses EMT and proliferative ability of CC cells via inhibiting the activation of the TGF-β/smad signaling pathway

**Fig. 8:**
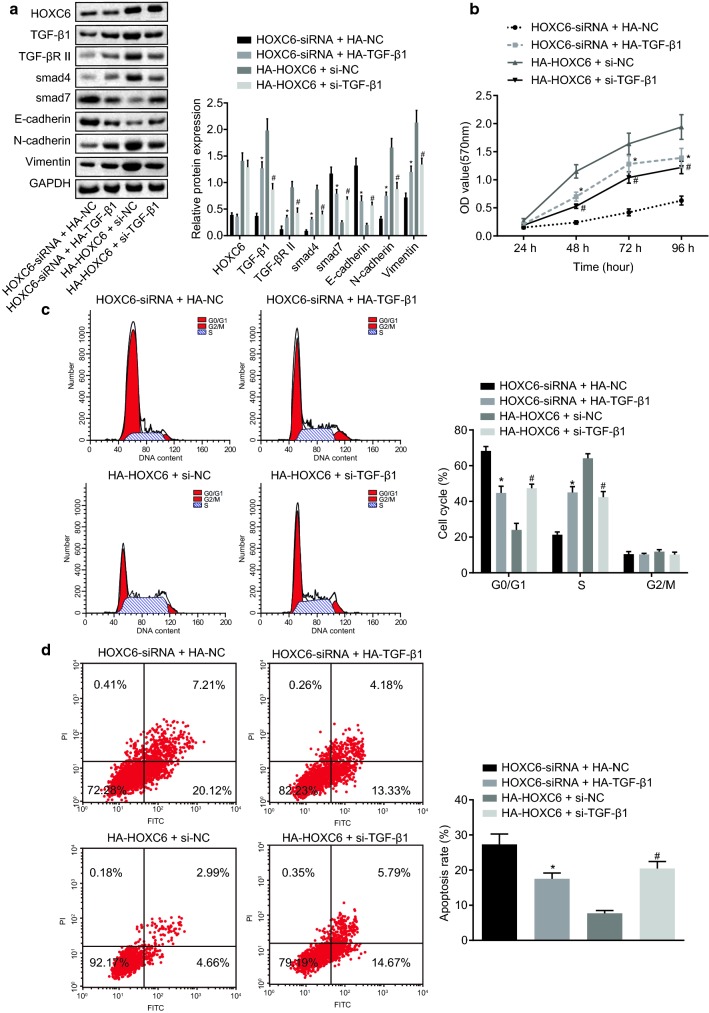
HOXC6 gene silencing inhibits EMT and proliferative ability of CC cells by suppressing the activation of the TGF-β/smad signaling pathway. **a** Protein expression of HOXC6, TGF-β1, TGF-β RII, smad4, smad7, E-cadherin, N-cadherin, and Vimentin determined by western blot analysis; **b** cell proliferation in each group after transduction; **c** cell cycle in each group after transduction; **d** cell apoptosis in each group after transduction; *p < 0.05, compared with the HOXC6-siRNA + HA-NC group; ^#^*p* < 0.05, compared with the HA-HOXC6 + si-NC group; HOXC6, homeobox C6; EMT, epithelial-mesenchymal transition; *CC* cervical carcinoma, *TGF-β* transforming growth factor-β, *smad* solvated metal atom dispersion, *NC* negative control

Based on HOXC6-siRNA, rescue assay was performed with the HA-TGF-β1 group (the overexpressed TGF-β1 plasmid) added. It was found that HOXC6-siRNA could inhibit EMT and proliferative ability of CC. After the treatment of HA-TGF-β1, EMT and proliferative ability of CC were rescued and the protein expression of the signaling pathway was also elevated, suggesting that HOXC6 worked via the TGF-β/smad signaling pathway. The HA-HOXC6 group presented enhanced EMT and proliferative ability of CC, which were diminished with the addition of si-TGF-β1 (Fig. 8a–d). These results provide evidence that HOXC6 gene silencing could inhibit the TGF-β/smad signaling pathway, thus suppressing EMT and proliferative ability of CC cells.

## Discussion

CC is the fourth commonest cancer in female worldwide [21]. Moreover, molecular mechanisms of CC remain poor understanding [22]. Furthermore, HOXC6 is involved in various cancers including lung cancer, osteosarcomas and breast cancer [10]. Therefore, in the current study, we focused on HOXC6 gene in regulation of CC progression with the involvement of TGF-β/smad signaling pathway, and our results demonstrate that HOXC6 gene silencing can suppress the EMT and cell viability in CC through inactivating the TGF-β/smad signaling pathway.

Initially, elevated mRNA and protein expression of HOXC6 were found in CC. HOXC6 gene is one of the homeoprotein transcription factors, which mainly regulate embryonic development and play roles in several cellular processes such as differentiation and morphogenesis [23]. Supportively, evidence has demonstrated that HOXC6 gene has association with various tumor progressions. For instance, overexpressed HOXC6 is frequently observed in prostate cancers and relates to adverse clinical parameters [24]. Overexpressed HOXC6 is closely linked to poor survival of patients with gastric cancer, and contributes to gastric carcinogenesis progression [25]. A previous study highlighted overexpression f HOXC6 in tumor tissues of colorectal cancer [26]. It has been reported that there are correlations of HOXC6 overexpression with increment of tumor stage, advanced nodal status in nasopharyngeal carcinoma [27].

Furthermore, the HOXC6-siRNA group witnessed a notable decline in the mRNA and protein expression of HOXC6, TGF-β1, TGF-β RII, smad4, smad7, N-cadherin and Vimentin, but an increase in E-cadherin expression. The TGF-β/smad signaling pathway can be initiated by TGF-β1 binding to TGF-β RII, and smads are essential intracellular effectors mediating TGF-β1 superfamily [28]. It has revealed that both smad4 and smad7 expression were related with Lauren classification and tumor differentiation, and smad4 expression was positively associated with TGF-β RII expression and negatively related to patients’ survival [29]. In osteosarcomas, the TGF-β superfamily seems to regulate the expression of HOXC6 [30]. At present, N-cadherin and E-cadherin, belonging to the classical cadherin family, are regarded as inducers of EMT [31], and Vimentin is widely considered as a marker of EMT [32]. EMT, a transdifferentiation process converting epithelial cells into motile mesenchymal cells, is indispensable in development and leads to cancer progression [33]. EMT confers epithelial cells with invasive capacity in a carcinoma, which is considered as a very critical phenomenon underlying metastatic dissemination [34] Previous evidence has suggested that EMT engages in primary tumor metastasis and reveals molecular mechanisms for CC metastasis [35]. Importantly, TGF-β and TGF-β-related proteins have been confirmed as the major inducers of EMT event in cancer development, and the convergence of TGF-β signaling pathways is essential for EMT [33, 36]. Based on these findings, it can be seen that HOXC6 gene silencing inhibit the EMT in CC by inactivating the TGF-β/smad signaling pathway.

Additionally, our study demonstrated that HOXC6 gene silencing inhibits cell proliferation and promotes cell apoptosis in CC cells. HOXC6 overexpression is capable of promoting colony formation and upregulating tumor growth factors, suggesting its important role in tumor growth [10]. It has revealed that HOXC6 overexpression induced colony formation and cell proliferation in gastric cancer [37]. Overexpressed HOXC6 is found in human head and neck squamous cell carcinoma cell lines and promote proliferation [38]. Consistently with our results, knockout of HOXC6 inhibited cell migration and proliferation in hepatocellular carcinoma [39]. HOXC6 gene silencing enables prostate cancer cell to apoptosis [40]. TGF-β family participated in the modulation of cell differentiation, proliferation, apoptosis, and motility, and TGF-β-induced growth inhibition was found to be repressed in tumor cells [41]. Previous research suggested that TGF-β/smad2/3 signaling pathway is a crucial regulator of cell apoptosis and proliferation of U251 cells [42]. What’s more, DKK3 overexpression suppressed collagen synthesis, induced cell apoptosis, and inhibited cell proliferation via TGF-β1/smad pathway in keloid fibroblasts [43]. These evidences support that HOXC6 gene silencing inhibited cell proliferation and promoted cell apoptosis in CC cells by inactivating the TGF-β/smad signaling pathway.

## Conclusion

Collectively, the key finding from this study is that HOXC6 gene can act as a regulator of CC cell viability. Suppression of HOXC6 gene results in suppressed cell proliferation, promoted cellular apoptosis and weakened abilities of EMT through blocking the activation of the TGF-β/smad signaling pathway. Our finding demonstrates the pivotal role of HOXC6 gene in CC advancement and suggests that it could serve as a therapeutic target in CC management, and further larger-scale studies and in vivo experiments are needed to validate this clinical utility.

## Abbreviations

HOXC6: Homeobox C6
CC: cervical carcinoma
EMT: epithelial-mesenchymal transition
TGF-β: transforming growth factor-β
AJCC: American Joint Committee on Cancer
TNM: tumor-node-metastasis
NBF: neutral buffered formalin
PBS: phosphate buffer solution
NC: negative control
DAB: diaminobenzidine
FBS: fetal bovine serum
DEPC: diethyl phosphorocyanidate
GAPDH: glyceraldehyde-3-phosphate dehydrogenase
PVDF: polyvinylidene fluoride
SDS-PAGE: sodium dodecyl sulfate polyacrylamide gel electrophoresis
TBST: tris buffered saline with Tween 20
HRP: horse radish peroxidase
ECL: enhanced chemiluminescence
DMSO: dimethyl sulphoxide
OD: optical density
PI: propidium iodide
EDTA: ethylene diamine tetraacetic acid
